# Association Between Body Mass Index and Hematotoxicity in Gynecological Cancer Patients Receiving Paclitaxel-Carboplatin Therapy: A Retrospective Study

**DOI:** 10.7759/cureus.99811

**Published:** 2025-12-22

**Authors:** Teruki Obana, Shinichiro Fujimoto, Kimiko Fujiwara, Manabu Takegami, Hidekatsu Nakai, Shozo Nishida, Noriaki Nagai, Masanobu Tsubaki

**Affiliations:** 1 Division of Pharmacy, Kindai University Hospital, Osaka, JPN; 2 Department of Pharmacy, Kindai University, Osaka, JPN; 3 Obstetrics and Gynecology, Kindai University Faculty of Medicine, Osaka, JPN; 4 Laboratory of Pharmacotherapy, Kagawa School of Pharmaceutical Sciences, Tokushima Bunri University, Kagawa, JPN

**Keywords:** body mass index: bmi, carboplatin, gynecological cancer, hematotoxicity, serum creatinine

## Abstract

Introduction: In gynecological cancer treatment, carboplatin dosage is generally calculated based on renal function to ensure appropriate drug exposure. In clinical practice, serum creatinine levels are sometimes adjusted when extremely low values are observed to avoid overdosing. Body mass index (BMI) has been suspected to influence the development of chemotherapy-induced hematotoxicity, but this relationship remains uncertain. This study aimed to investigate the effect of BMI on hematotoxicity in gynecological cancer patients receiving paclitaxel-carboplatin combination therapy (TC therapy).

Methods: Using electronic medical records, patients with gynecological cancer who received TC therapy for the first time between January 2014 and December 2021 were included in this retrospective study. BMI, Cockcroft-Gault formula (C-G formula) factors such as age, body weight, serum creatinine (sCr), and laboratory values related to hematotoxicity (leukopenia, anemia, neutropenia, and thrombocytopenia) prior to and after TC therapy. Nadir values obtained before the start of the second course of TC therapy were extracted from the electronic medical records. Among the extracted patient information, the presence or absence of grade ≥ 3 hematotoxicity was used as the outcome variable, and the grade of each blood cell prior to TC therapy, age, glomerular filtration rate (GFR) calculated using the C-G formula, and BMI were used as explanatory variables in multiple logistic regression analysis.

Results: Among the 273 eligible patients, no significant correlation was found between grade ≥ 3 hematotoxicity and BMI (leukopenia: odds ratio (OR) = 1.08, 95% confidence interval (CI) = 0.98-1.19, p = 0.12; anemia: OR = 1.06, 95% CI = 0.85-1.30, p = 0.57; neutropenia: OR = 1.05, 95% CI = 0.97-1.15, p = 0.20; thrombocytopenia: OR = 1.34, 95% CI = 0.93-1.89, p = 0.10). Younger age was associated with an increased risk of grade ≥ 3 leukopenia (OR = 0.96, 95% CI = 0.92-1.00, P = 0.035), and lower GFR was associated with increased risks of grade ≥ 3 leukopenia (OR = 0.97, 95% CI = 0.95-1.00, P = 0.049) and anemia (OR = 0.95, 95% CI = 0.89-0.99, P < 0.01).

Conclusions: BMI was not associated with grade ≥ 3 hematotoxicity across all hematologic endpoints. Although serum creatinine correction to 0.7 mg/dL may reduce BMI-related variability in renal function estimation, this interpretation remains speculative because our study did not include a non-corrected comparison group.

## Introduction

Carboplatin, a platinum-containing compound, was developed to reduce the side effects of cisplatin, such as renal impairment and vomiting [1]. Compared with cisplatin, carboplatin causes less kidney damage and therefore does not require fluid balance management such as rehydration and infusions [2]. However, hematotoxicity appears more frequently with carboplatin than with cisplatin, and supportive care is implemented according to the patient’s symptoms. Carboplatin has an elimination clearance strongly correlated with the glomerular filtration rate (GFR), and its hematotoxicity is correlated with the area under the blood concentration-time curve (AUC) after administration [3,4]. Therefore, the carboplatin dosage is generally determined from GFR and AUC values (Calvert formula: $\mathrm{Carboplatin \, dosage} = \mathrm{target \, AUC} \times (\mathrm{GFR} + 25)$) [5]. At our hospital, GFR is substituted for creatinine clearance (CLCr) calculated by the Cockcroft-Gault formula (C-G formula: $\mathrm{CLCr} \, (\mathrm{mL/min}) = \frac{(140 - \mathrm{age}) \times \mathrm{body\ weight} \, (\mathrm{kg}) \times 0.85}{72 \times \mathrm{sCr} \, (\mathrm{mg/dL})}$) [6].

To prevent carboplatin overdosage in gynecological oncology, it is recommended to determine the carboplatin dose based on the Gynecologic Oncology Group/National Comprehensive Cancer Network (GOG/NCCN) guidelines, which specify correcting serum creatinine (sCr) to 0.7 mg/dL if sCr < 0.7 mg/dL [7]; this protocol has been implemented in our hospital. Body mass index (BMI) in carboplatin-treated patients might affect hematotoxicity [8-10]; however, the results regarding the relationship between BMI and hematotoxicity are not uniform among previous studies due to different methods of determining the carboplatin dosages. In addition, a previous study reported a positive correlation between CLCr and BMI [11], but the relationship between BMI and hematotoxicity in carboplatin-treated patients using CLCr corrected for sCr has not been elucidated. We hypothesized that correction of sCr to 0.7 mg/dL would reduce BMI-related differences in estimated renal function and thereby attenuate BMI-associated variability in hematotoxicity. The primary objective of this study was to evaluate the association between BMI and the development of grade ≥ 3 hematotoxicity during the first cycle of paclitaxel-carboplatin combination therapy (TC therapy). The secondary objectives were to assess whether age and GFR were associated with hematotoxicity under this dosing strategy.

## Materials and methods

Study design and patients

Patients diagnosed with ovarian, cervical, uterine, or other gynecological cancers at the Department of Gynecology of our hospital who also received TC therapy for the first time between January 2014 and December 2021 were included in this retrospective study. All patients received three weekly TC therapies consisting of paclitaxel (180 mg/m²) in combination with carboplatin dosed according to the Calvert formula. No patients received granulocyte colony-stimulating factor prophylactically prior to TC therapy or after the first cycle. The exclusion criteria were as follows: patients in whom 24-hour urine storage was used to calculate CLCr values, patients with unclear sCr values used in the calculation of the carboplatin dosage, those with unclear nadir values, and patients with hematological disorders that could confound the evaluation of hematotoxicity.

Sample size

The number of cases was determined based on the proportion of grade ≥ 3 hematotoxicities previously reported in cervical cancer patients treated with TC therapy (neutropenia: 76.2%, anemia: 44.4%, thrombocytopenia: 24.6%) [12]. The event per variable method was applied to determine the minimum required number of events [13]. Since we planned to set four explanatory variables, a minimum of 4 × 10 events was required. Furthermore, if we estimate the necessary sample size based on thrombocytopenia, which is the least common adverse event in the above percentages, the calculation is as follows:

$\frac{40}{0.246} \approx 163$ cases

**Figure 1:**
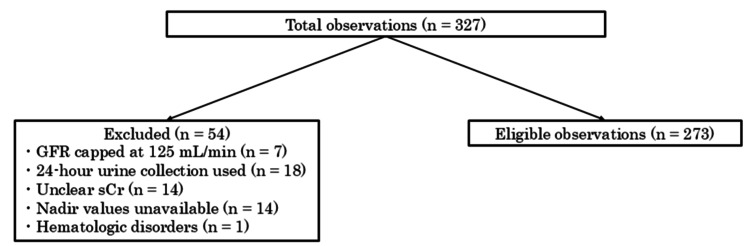
Flow diagram of patient selection and exclusion. Of the 327 initially screened observations, 54 were excluded for the following reasons: GFR capped at 125 mL/min (n = 7), use of 24-hour urine collection for renal function assessment (n = 18), unclear serum creatinine values (n = 14), unavailable nadir hematologic values (n = 14), and pre-existing hematologic disorders (n = 1). The remaining 273 observations were included in the final analysis. GFR: glomerular filtration rate; sCr: serum creatinine.

Based on statistical evidence, 327 cases of TC therapy administered for the first time within the target period were extracted from the prior drug usage records. Of these, cases that met the inclusion criteria and did not meet any exclusion criteria were included in the study (Figure 1).

Calculation of the carboplatin dosage

CLCr values were calculated using the C-G formula: sCr = 0.7 mg/dL was used when sCr ≤ 0.7 mg/dL, and the measured value was used when sCr > 0.7 mg/dL. The following formula was used to calculate CLCr, since this study was conducted on gynecological cancers, and all patients were female individuals:

$\mathrm{CLCr} \, (\mathrm{mL/min}) = \frac{(140 - \mathrm{age}) \times \mathrm{actual\ body\ weight} \, (\mathrm{kg}) \times 0.85}{72 \times \mathrm{sCr} \, (\mathrm{mg/dL})}$

The Calvert formula was used to calculate the carboplatin dosage, and the GFR values were substituted with CLCr. In this study, patients with a GFR ≥ 125 mL/min were excluded; therefore, all carboplatin dose calculations were performed within this range. According to the Calvert formula, a GFR of 125 mL/min corresponds to a calculated dose of $\mathrm{AUC} \times (125 + 25) = \mathrm{AUC} \times 150$.

$\mathrm{Carboplatin \, dosage} = \mathrm{target \, AUC} \times (\mathrm{GFR} + 25)$

($\mathrm{Maximum \, dosage} = \mathrm{target \, AUC} \times 150 \, \mathrm{mL/min}$) [7,14].

Data collection

Patient background characteristics, physical examination results, blood laboratory values, and carboplatin dosage were obtained from patients’ electronic medical records. Blood test data for sCr, CLCr, leukocytes, hemoglobin, neutrophils, and platelets were extracted. Blood test values prior to TC therapy and nadir values observed during the first cycle (i.e., before the initiation of the second cycle) were included to confirm the occurrence of hematotoxicity after completion of the first course of TC therapy. The nadir values for hematotoxicity were primarily assessed using blood tests performed on day 14 after the first cycle of TC therapy. In cases where blood sampling was conducted on days other than day 14 due to patient scheduling constraints, the lowest recorded value during the post-treatment period was considered the nadir. Hematotoxicity was evaluated on the basis of the Common Terminology Criteria for Adverse Events (CTCAE) version 5.0 [15]. Grade ≥3 hematotoxicity was selected as the outcome because such severe events generally warrant treatment modification, including dose reduction, treatment delay, or temporary discontinuation in clinical practice.

Statistical analysis

Among the extracted patient information, the presence or absence of grade ≥ 3 hematotoxicity was used as the outcome variable, and multiple logistic regression analysis was used with the grade of each hematotoxicity prior to TC therapy, age, GFR values used in the C-G formula, and BMI as explanatory variables. Multicollinearity among explanatory variables was assessed using variance inflation factors (VIFs), and all VIF values were < 5. Statistical analyses were performed using JMP (version 17; SAS Institute, Cary, NC, USA), with P < 0.05 considered statistically significant.

Ethics statements

This study was conducted in accordance with the tenets of the Declaration of Helsinki and ethical guidelines for clinical research and was approved by the Ethics Committee, Kindai University School of Medicine (approval number: R06-114). It was an observational study using existing data. The study was anonymized to prevent the identification of specific individuals. Since there were no direct study participants to whom the study could be explained, no explanatory documents or consent forms were prepared. Instead, all subjects were provided with an opt-out option. Furthermore, this study complied with all applicable local privacy and data protection regulations.

## Results

Patients’ baseline characteristics

A total of 273 patients were included in the study, and their baseline characteristics are summarized in Tables 1-2. Among these patients, 216 (79.1%) had sCr values < 0.7 mg/dL, and their sCr values were corrected to 0.7 mg/dL for the calculation of CLCr. These CLCr values were then used in place of GFR when determining the carboplatin dosage.

**Table 1:** Patients’ baseline characteristics. BMI: body mass index, BSA: body surface area, GFR: glomerular filtration rate, Pre: prior to treatment, Post: after treatment, WBC: white blood cell, NEUT: neutrophil, RBC: red blood cell, Hb: hemoglobin, PLT: platelet.

Variable	Median (range)
Age (years)	58 (31–86)
Height (cm)	156.4 (135.0–173.0)
Weight (kg)	53.9 (31.8–94.8)
BMI (kg/m^2^)	21.6 (14.0–38.0)
BSA (m^2^)	1.531 (1.143–1.952)
Paclitaxel dosage (mg)	274.3 (182.0–351.0)
Carboplatin dosage (mg)	580.0 (256.0–870.4)
GFR (mL/min)	72.1 (17.8–120.2)
Pre WBC (×10³/µL)	5.66 (2.28–20.30)
Post WBC (×10³/µL)	2.80 (0.29–9.90)
Pre NEUT (×10³/µL)	3.61 (0.37–13.91)
Post NEUT (×10³/µL)	1.08 (0.03–8.20)
Pre RBC (×10⁶/µL)	3.96 (2.16–6.22)
Post RBC (×10⁶/µL)	3.81 (1.71–5.13)
Pre Hb (g/dL)	11.60 (6.90–15.00)
Post Hb (g/dL)	11.20 (7.20–14.50)
Pre PLT (×10^4^/µL)	28.10 (4.30–77.30)
Post PLT (×10^4^/µL)	20.40 (2.30–61.60)

**Table 2:** Clinical background and carboplatin dosing characteristics. Data are presented as number (percentage). Total n = 273. sCr: serum creatinine, AUC: area under the curve, BMI: body mass index. BMI categories were defined according to the World Health Organization classification.

Variable	n (%)	
Ovarian cancer		120 (44.0)
Cervical cancer		59 (21.6)
Uterine cancer		89 (32.6)
Others		5 (1.8)
sCr = 0.7 mg/dL		216 (79.1)
sCr > 0.7 mg/dL		57 (20.9)
AUC = 6		272 (99.6)
AUC = 5		1 (0.4)
Underweight: BMI < 18.5 kg/m^2^		42 (15.4)
Normal: BMI 18.5–24.9 kg/m^2^		167 (61.2)
Overweight: BMI 25–29.9 kg/m^2^		42 (15.4)
Obese: BMI ≥ 30 kg/m^2^		22 (8.0)

Hematotoxicity

The grades of hematotoxicity prior to and after TC therapy are shown in Table 3. Among the 273 patients, grade ≥3 neutropenia was the most frequent hematotoxicity (46.5%), followed by leukopenia (16.8%), anemia (3.7%), and thrombocytopenia (1.5%).

**Table 3:** Effects of TC therapy on hematotoxicity. TC therapy: paclitaxel-carboplatin combination therapy (TC therapy); Pre: prior to treatment; Post: after treatment; Grades were assessed according to Common Terminology Criteria for Adverse Events (CTCAE) version 5.0 [15].

Hematotoxicity and grade	Pre (n)	Post (n)
Leukopenia grade 0	263	95
Leukopenia grade 1	5	20
Leukopenia grade 2	5	112
Leukopenia grade 3	0	41
Leukopenia grade 4	0	5
Neutropenia grade 0	256	51
Neutropenia grade 1	14	39
Neutropenia grade 2	2	56
Neutropenia grade 3	0	66
Neutropenia grade 4	1	61
Anemia grade 0	137	110
Anemia grade 1	97	111
Anemia grade 2	37	42
Anemia grade 3	2	10
Anemia grade 4	0	0
Thrombocytopenia grade 0	267	203
Thrombocytopenia grade 1	5	62
Thrombocytopenia grade 2	0	4
Thrombocytopenia grade 3	1	3
Thrombocytopenia grade 4	0	1

Association between hematotoxicity and patient background characteristics

Multiple logistic regression analysis was used to analyze the nadir values after TC therapy as the outcome variable and factors influencing patient background characteristics (GFR, BMI, age, and grade of hematotoxicity prior to TC therapy) in terms of the occurrence of grade ≥ 3 hematotoxicity as explanatory variables, and the results are shown in Tables 4-7. No significant association was found between BMI and any grade ≥ 3 hematotoxicity outcome (leukopenia: odds ratio (OR) = 1.08, 95% confidence interval (CI) = 0.98-1.19, p = 0.12; anemia: OR = 1.06, 95% CI = 0.85-1.30, p = 0.57; neutropenia: OR = 1.05, 95% CI = 0.97-1.15, p = 0.20; thrombocytopenia: OR = 1.34, 95% CI = 0.93-1.89, p = 0.10). Because only a very small number of patients developed thrombocytopenia, the statistical power to detect associations for this endpoint was limited. Additionally, the following variables were statistically significant. Age was significantly associated with grade ≥ 3 leukopenia (OR = 0.96, 95% CI = 0.92-1.00, P = 0.035). Similarly, lower GFR was significantly associated with increased risks of grade ≥ 3 leukopenia (OR = 0.97, 95% CI = 0.95-1.00, P = 0.049) and anemia (OR = 0.95, 95% CI = 0.89-0.99, P < 0.01). Model fit was evaluated using Akaike’s information criterion (AIC) and the Hosmer-Lemeshow goodness-of-fit test. Adequate calibration was observed in the models for anemia (p = 1.000), leukopenia (p = 0.913), and thrombocytopenia (p = 1.000). However, the neutropenia model showed a lack of fit (p = 0.0001), and therefore its results should be interpreted with caution. The corresponding AIC values were 63.48 (anemia), 248.52 (leukopenia), 40.82 (thrombocytopenia), and 374.71 (neutropenia). This lack of fit may be attributable to the high event rate of neutropenia and the limited variability of covariates for this outcome.

**Table 4:** Risk factors for leukopenia (grade ≥ 3). Significance was determined using multiple logistic regression analysis. *P < 0.05. CI: confidence interval; GFR: glomerular filtration rate; BMI: body mass index; χ²: chi-square statistic.

Factor	Odds ratio	95% CI	χ^2^	P-value
Leukopenia prior to chemotherapy	-	-	5.97	0.051
Age	0.96	0.92–1.00	4.45	0.035*
GFR	0.97	0.95–1.00	3.86	0.049*
BMI	1.08	0.98–1.19	2.43	0.12

**Table 5:** Risk factors for anemia (grade ≥ 3). Significance was determined using multiple logistic regression analysis. *P < 0.05. CI: confidence interval; GFR: glomerular filtration rate; BMI: body mass index; χ²: chi-square statistic.

Factor	Odds ratio	95% CI	χ^2^	P-value
Anemia prior to chemotherapy	-	-	30.80	<0.01*
Age	0.97	0.89–1.04	0.683	0.41
GFR	0.95	0.89–0.99	5.35	0.021*
BMI	1.06	0.85–1.30	0.32	0.57

**Table 6:** Risk factors for neutropenia (grade ≥ 3). Significance was determined using multiple logistic regression analysis. *P < 0.05. CI: confidence interval; GFR: glomerular filtration rate; BMI: body mass index; χ²: chi-square statistic.

Factor	Odds ratio	95% CI	χ^2^	P-value
Neutropenia prior to chemotherapy	-	-	15.20	< 0.01*
Age	0.99	0.96–1.02	0.42	0.52
GFR	1.00	0.97–1.02	0.29	0.59
BMI	1.05	0.97–1.15	1.68	0.20

**Table 7:** Risk factors for thrombocytopenia (grade ≥ 3). Significance was determined using multiple logistic regression analysis. No variables reached statistical significance (p > 0.05). CI: confidence interval; GFR: glomerular filtration rate; BMI: body mass index; χ²: chi-square statistic.

Factor	Odds ratio	95% CI	χ^2^	P-value
Thrombocytopenia prior to chemotherapy	-	-	5.23	0.073
Age	0.85	0.71–1.01	3.25	0.071
GFR	1.00	0.95–1.11	0.01	0.94
BMI	1.34	0.93–1.89	2.67	0.10

## Discussion

Under the specific dosing strategy applied in this study (namely, sCr correction to 0.7 mg/dL and Calvert formula-based carboplatin dosing) BMI was not associated with grade ≥ 3 hematotoxicity. Because sCr values < 0.7 mg/dL were corrected and CLCr was substituted for GFR in dose calculation, BMI-related differences in estimated renal function may have been attenuated. BMI may show different associations under alternative dosing strategies. This correction prevents renal function overestimation in individuals with low muscle mass, thereby standardizing carboplatin exposure. Given that BMI was not a major determinant under this dosing strategy, we next examined other clinical factors associated with hematotoxicity.

Younger age was associated with a higher likelihood of developing grade ≥ 3 leukopenia, while lower GFR was associated with increased risks of grade ≥ 3 leukopenia and grade ≥ 3 anemia. Grade ≥ 3 anemia was also significantly associated with baseline anemia prior to chemotherapy. Grade ≥ 3 neutropenia showed a significant association with baseline neutropenia prior to chemotherapy; however, no significant associations were observed with age or GFR. No significant associations were observed for grade ≥ 3 thrombocytopenia. From a clinical standpoint, these results suggest the need for closer hematologic monitoring in patients with reduced renal function or younger age, who may be more susceptible to severe cytopenias during TC therapy. In addition, the observed association between younger age and a higher risk of leukopenia may be partly explained by pharmacokinetic considerations. Younger patients generally have higher GFR values, resulting in larger carboplatin doses when the Calvert formula is applied. Moreover, younger individuals tend to maintain better performance status and are thus less likely to undergo dose reductions, which may lead to greater cumulative exposure. These mechanisms may help explain why younger patients exhibited a higher likelihood of leukopenia in our cohort. However, unmeasured confounding factors cannot be fully excluded.

Table 4 shows the significant correlation between grade ≥ 3 leukopenia and age. Several reports have shown no significant difference in hematotoxicity between older and younger patients when carboplatin-containing regimens were administered [16,17]. However, these reports differ in the manner in which the carboplatin dosages were determined. Additionally, there was no significant difference in hematotoxicity in patients with advanced ovarian cancer treated with TC therapy between patients older than 70 years of age and those younger than 70 years of age [18]. Although previous studies have reported no significant differences in hematotoxicity between older and younger patients, our analysis indicated that younger patients had a higher risk of developing grade ≥ 3 leukopenia than older patients.

Significant negative correlations were found between GFR and grade ≥ 3 leukopenia (Table 4) and between GFR and anemia (Table 5). Previous studies reported that CLCr calculated using the Jelliffe formula is not a predictor of hematotoxicity [14]. However, Hurria et al. reported that a CLCr < 34 mL/min, calculated using the Jelliffe equation, is a risk factor for chemotherapy toxicity in patients older than 65 years of age [19]. This study finding suggests that decreasing GFR was associated with an increased risk of grade ≥ 3 leukopenia and anemia in patients receiving TC therapy.

Because age is a component of the C-G formula used to estimate renal function, age and GFR are inherently correlated. Our results suggested that younger age was associated with an increased risk of leukopenia. Additionally, lower GFR was associated with increased risks of leukopenia and anemia. Both variables were retained in the multivariable model because they represent clinically distinct concepts, that is, chronological age and renal function. However, their independent effects should be interpreted cautiously due to their mathematical interdependence. The potential multicollinearity between age and GFR, which are mathematically related through the C-G formula, may limit the interpretability of their independent associations in our analysis. Furthermore, although these associations reached statistical significance, the effect sizes were small (odds ratios close to 1.0), indicating that the clinical impact may be limited and should be interpreted cautiously. Taken together, these findings suggest that although age and renal function were statistically associated with hematotoxicity, their effects were modest and may reflect dosing-related factors rather than independent biological risk factors.

Additionally, baseline hematologic status warrants consideration. Table 5 shows that anemia prior to TC therapy was significantly correlated with the occurrence of grade ≥ 3 anemia. The baseline hemoglobin level prior to chemotherapy may be a risk factor for inducing chemotherapy-induced anemia [20]. In this study, as in previous reports, anemia prior to TC therapy was shown to be a risk factor for grade ≥ 3 anemia during treatment [21]. Anemia occurs frequently in patients with lung cancer and gynecological cancer, who are more likely to receive platinum-containing regimens [22]. Additionally, 49% of women with gynecological cancers reported being anemic at diagnosis [23]. In this study, 49.8% of the patients were anemic prior to TC therapy, similar to that reported in a previous study, and this may have contributed to subsequent hematotoxicities. Previous studies in gynecological cancers have reported that pretreatment anemia is a risk factor for chemotherapy-induced hematotoxicities, including febrile neutropenia (FN) [24]. Although FN is a different endpoint than neutropenia, these findings suggest that anemia prior to TC therapy may predispose patients to severe myelosuppression.

In Table 7, no significant correlation was found between thrombocytopenia prior to TC therapy and grade ≥ 3 thrombocytopenia; however, this may be due to the small number of patients who had grade ≥ 1 thrombocytopenia prior to TC therapy. Thrombocytopenia is a dose-limiting toxicity of carboplatin and requires further investigation.

With respect to institutional dosing strategies, Imamura et al. reported that nearly half of gynecological institutions in Japan use CLCr calculated by the C-G formula as the GFR in the Calvert formula to determine the carboplatin dosage [25]. The sCr level can differ depending on the assay method. In Japan, the enzymatic method is commonly used, whereas in the United States, the isotope dilution mass spectrometry (IDMS) method is widely adopted. Because the IDMS method yields lower sCr values than the enzymatic method, substitution of IDMS-based values into the Calvert formula may result in an overestimation of CLCr and overdosing of carboplatin [26]. Therefore, the National Cancer Institute/Cancer Therapy Evaluation Program recommends a maximum GFR of 125 mL/min when calculating carboplatin doses (e.g., AUC 6 = 900 mg, AUC 5 = 750 mg) [7]. Because carboplatin dosing directly depends on the estimated GFR, different estimation formulas (such as Modification of Diet in Renal Disease (MDRD) and Chronic Kidney Disease Epidemiology Collaboration (CKD-EPI) equations) could yield different renal function values and consequently alter the observed associations with hematotoxicity.To address discrepancies in renal function estimation, some institutions in Japan have also set upper limits to avoid overdose. The dosage of anticancer drugs is often calculated on the basis of body weight and body surface area, and there is concern that patients with obesity may experience increased side effects due to higher dosages. According to the American Society of Clinical Oncology (ASCO), actual body weight should be used when calculating chemotherapy doses, even in patients with obesity [27]. In contrast, NRG Oncology recommends the use of adjusted body weight for patients with a BMI ≥ 25 kg/m2 when dosing carboplatin [14].This discrepancy in guidelines highlights the ongoing debate regarding the optimal dosing strategy for patients with obesity, and it is particularly relevant to our study population, in which BMI varied widely. Taken together, these findings provide a practical context for interpreting differences among international dosing recommendations. ASCO endorses the use of actual body weight for chemotherapy and carboplatin dosing, whereas NRG Oncology recommends adjusted body weight for patients with higher BMI. Our results, showing no association between BMI and the development of grade ≥ 3 hematotoxicity and highlighting renal function as a stronger determinant, align more closely with the rationale behind the NRG Oncology approach [14]. These observations suggest that dosing strategies primarily based on renal function, rather than body size alone, warrant further evaluation in real-world chemotherapy settings. Although this study did not examine the correlation between paclitaxel toxicity and BMI, several previous studies have reported that no correlation was found between them [28,29]. According to the ASCO, actual body weight should be used when estimating GFR for carboplatin dosing, even in patients with obesity [27]. In this study, a carboplatin dosage based on the NRG Oncology guidelines was performed, and the results suggest that determining the carboplatin dosage by correcting for the sCr and using the Calvert formula is useful for eliminating the influence of BMI differences on the development of hematotoxicity. Accordingly, sCr correction and Calvert formula-based dosing may represent a reasonable approach to standardizing carboplatin dosing, although prospective validation is required. Although additional validation is needed, this approach may support more consistent and clinically appropriate dosing across patients with differing body sizes.

This study has some limitations. First, all patients with sCr < 0.7 mg/dL had their values corrected to 0.7 mg/dL, which precluded direct comparison between patients with and without correction; therefore, the mitigating effect of this correction on BMI-related hematotoxicity should be interpreted with caution. In addition, sCr and estimated GFR values prior to correction were not retained in the database, and the impact of sCr correction on the distribution of estimated GFR could not be quantitatively evaluated. Although sCr values were corrected to a minimum of 0.7 mg/dL in accordance with the NRG Oncology guidelines, this approach does not account for individual differences in creatinine production. As renal function was estimated solely from sCr, the accuracy of renal function assessment may vary among patients. Second, this was a single-center retrospective study; therefore, the level of evidence was lower than that of prospective studies. Future multicenter studies including more diverse patient populations and comparisons of different GFR estimation formulas may help clarify whether BMI modifies hematotoxicity under various dosing strategies. Third, BMI was used as an anthropometric measure; however, BMI does not distinguish between fat mass and lean mass. Differences in body composition may influence carboplatin distribution and clearance more directly than BMI alone. Information on potential confounders, such as nutritional status and the etiology of pre-existing anemia, was inconsistently documented in the medical records and could not be systematically evaluated because of the retrospective design. Regression analyses were conducted using available-case data without imputation; thus, missing or incomplete records did not directly influence the construction of the multivariable regression models, although residual confounding cannot be excluded. Fourth, hematotoxicity was evaluated only during the first cycle of TC therapy, and changes in laboratory values over subsequent cycles could not be adequately assessed. As TC therapy is generally administered over multiple cycles, cumulative hematotoxicity and long-term renal effects may not have been fully captured in this analysis. A previous study reported that the risk of renal dysfunction increases with long-term carboplatin administration [30], and further investigation is warranted. Finally, although carboplatin dosing was calculated using a target AUC of 6 in most patients and an AUC of 5 in one patient, actual pharmacokinetic exposure may differ from the intended AUC because pharmacokinetic measurements were not obtained. Consequently, the association between true drug exposure and hematotoxicity could not be directly evaluated. As this was a single-center study conducted in a Japanese clinical setting, the generalizability of the findings to other populations with different body composition profiles may be limited. Continued accumulation of evidence through multicenter prospective studies is therefore warranted.

## Conclusions

In this retrospective study of gynecological cancer patients receiving paclitaxel-carboplatin therapy, BMI was not associated with the development of grade ≥ 3 hematotoxicity during the first treatment cycle under a dosing strategy incorporating sCr correction to 0.7 mg/dL and Calvert formula-based carboplatin dosing. Across all hematologic endpoints, the odds ratios for BMI were close to 1.0 with narrow confidence intervals, indicating no clinically meaningful association. Although age and GFR showed statistically significant associations with certain hematotoxicities, the effect sizes were small and should be interpreted cautiously, particularly given their mathematical interdependence within the Cockcroft-Gault formula. These findings suggest that, within this specific dosing framework, BMI alone may not be a major determinant of severe hematotoxicity; however, prospective multicenter studies incorporating alternative renal function estimation methods and cumulative toxicity assessment are warranted to confirm generalizability.
